# When neuro-robots go wrong: A review

**DOI:** 10.3389/fnbot.2023.1112839

**Published:** 2023-02-03

**Authors:** Muhammad Salar Khan, James L. Olds

**Affiliations:** Interdisciplinary Neuroscience Program, Center for Biomedical Science and Policy, Schar School of Policy and Government, George Mason University, Arlington, VA, United States

**Keywords:** neuro-robotic systems, explainability, explainable AI (X-AI), neuro-robotic failures, explainable neuro-robots, neuro-robotic models, responsible neuro-robots

## Abstract

Neuro-robots are a class of autonomous machines that, in their architecture, mimic aspects of the human brain and cognition. As such, they represent unique artifacts created by humans based on human understanding of healthy human brains. European Union’s Convention on Roboethics 2025 states that the design of all robots (including neuro-robots) must include provisions for the complete traceability of the robots’ actions, analogous to an aircraft’s flight data recorder. At the same time, one can anticipate rising instances of neuro-robotic failure, as they operate on imperfect data in real environments, and the underlying AI behind such neuro-robots has yet to achieve explainability. This paper reviews the trajectory of the technology used in neuro-robots and accompanying failures. The failures demand an explanation. While drawing on existing explainable AI research, we argue explainability in AI limits the same in neuro-robots. In order to make robots more explainable, we suggest potential pathways for future research.

## 1. Introduction

**FIGURE 1:**
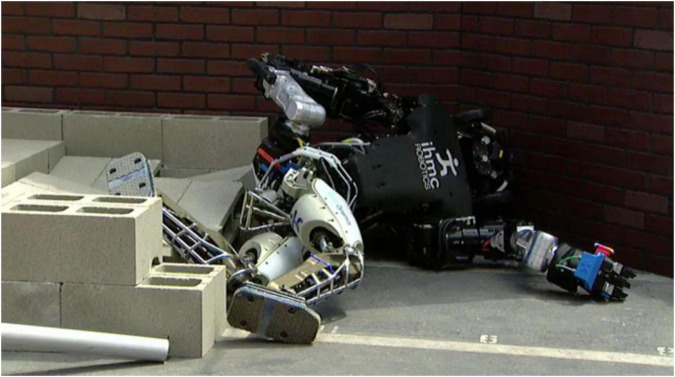
IHMC’s Atlas was one of the robots that fell during the DARPA robotics challenge finals. Source: DARPA (Guizzoevan and Ackerman, 2015).

Japan’s Henna Hotel (literally translates in English to “strange hotel”)—opened in 2015 with a staff of 243 robots—has cut its robotic workforce, stating that the robots started annoying the guests frequently (Hertzfeld, 2019). The firing of the robots comes after many objections from both staff and customers. Many of these robots created more work for the hotel staff instead of reducing it. There are numerous other instances of robot failures. In the famous DARPA Robotics Challenge, many robots fell over (including the IHMC’s Atlas robot shown in Figure 1), and some fell over multiple times (Guizzoevan and Ackerman, 2015).

Beyond traditional robotic failures, early-stage neuro-robots employing embodied intelligence—notably humanoid (developmental) robots inspired by the human nervous system and most visually resembling humans—also failed in several instances, particularly in unpredictable situations. For example, Pepper, a humanoid robot from the Japanese firm SoftBank, could not deliver at jobs it was designed for, from entertaining residents at nursing homes to welcoming customers in banks (Ryan, 2021). Similarly, the Atlas humanoid robot developed by Boston Dynamics failed while doing parkour (Pescovitz, 2021). Many other humanoids that closely approximate neuro-robots include: the H-7 of the University of Tokyo (Nishiwaki et al., 2007), Asimo of Honda Motor Corporation (ASIMO, 2011), Qrio of Sony Corporation (Carnegie Mellon Today, 2005), DB from Utah-based company Sarcos (Cheng, 2015), or HRP-2 from the Japanese Humanoid Robotics Program (Hirukawa et al., 2004), among others. Although representing high technological achievements, these robots have yet to achieve high success and complete precision, for example, in terms of behavioral diversity and dexterity, as seen in humans. This is because they are not ready to exploit their system-environment interaction to a higher degree and are yet to fully develop in handling unplanned real-life situations (Pfeifer and Bongard, 2007).

Aside from lab competitions, experiments, and toy models, robots are currently deployed in several fields ranging from education, health, and agriculture to transport, hospitality, tourism, and the food industry. Technological capabilities, including robots, are expected to stimulate speedy process automation, improved service delivery, enhanced productivity and efficiency, advanced technological innovation and leadership, and higher economic growth (Romer, 1990; Khan, 2022). Several ethical and legal issues, such as control, autonomy, and regulation, concern the development and design of neuro-robots (Nyholm, 2022), but here we focus on neuro-robotic failures. As neuro-robots get deployed, neuro-robotic failures would cause severe repercussions to human counterparts in all consequential domains. Therefore, understanding how such robotic decisions are implemented is crucial in avoiding current and future neuro-robotic failures. What makes them go wrong? Why do neuro-robots decide in a particular manner? Why were they “too annoying” for the guests in the Hotel? What makes humanoid robots fail? These and many other questions merit our attention toward designing and exploring the issue of explainable robots.

Neural-inspired robots that deploy artificial intelligence (AI) and embodied intelligence (i.e., neuro-robots) need to be explained further. Situated in a natural environment, sensing their environment and acting on it, neuro-robots have control systems based on the principles of nervous systems (Krichmar, 2008). We would assume that robots (and neuro-robots) have become more capable than ever before because the cost of sensors has reduced significantly, and AI algorithms have matured exponentially (Silver et al., 2016). Yet, most AI algorithms, particularly the Blackbox deep neural networks, are still largely unexplainable (de Bruijn et al., 2022; Khan et al., 2022). Similarly, many brain processes are yet to be unknotted, not only in humans but also in animals with simple brains (Krichmar et al., 2019). Hence what follows is that the neuro-robots designed, taking into account sophisticated AI algorithms and human brain mimicry, are, to a large extent, unexplainable.

Explaining and understanding the human brain and how neural activity gives rise to behavior is a permanent subject of discussion and learning in the realm of neuroscience (Ungerleider and Haxby, 1994; Eickhoff et al., 2018; Vijayakumar et al., 2018; Klein et al., 2019; Chen K. et al., 2020). But in the case of AI, European Union (EU) law warrants AI to provide an immediate explanation as required by the “Right to Explanation” enacted in 2016, specifically in the context of adverse decisions affecting Europeans (ITIF, 2018). A neuro-robotic explanation is even more desirable in light of the EU Convention on Roboethics 2025 (Enlightenment of an Anchorwoman, 2010), the Institute of Electrical and Electronics Engineers (IEEE) Guideline for ethical designs 2019 (Bulan, 2019), and Japan’s robotics principles (Pepito et al., 2019) inspired by Isaac Asimov’s Three Laws of Robotics aiming to protect humans interacting with robots (Anderson, 2007). Furthermore, the latest push toward designing explainable and “responsible” automated systems comes from the White House Office of Science and Technology Policy (OSTP) in the US (The White House OSTP, 2022).

To explain a bit more, the EU’s Convention requires that the design of all robots (including neuro-robots) must include provisions for the complete traceability of the robots’ actions. Furthermore, the IEEE Guideline for Ethically Aligned Design asks that robot decisions must be transparent and supported by clear reasoning. Similarly, the need for explainability indirectly flows from Japan’s “Ten Principles of Robot Law,” which requires robots to serve humankind and robotic manufacturers to be held responsible for their creation. Finally, the recent passage of “Blueprint for an AI Bill of Rights” by the White House OSTP in the US also outlines crucial principles that should lead the design, application, and deployment of automated systems (including neuro-robots) to protect the US citizens in the age of AI (The White House OSTP, 2022).

While most of these policies (and guidelines) talk about general AI and robotics, the extent that neuro-robots are automated systems, they fall under the realm of these policies. However, neuro-robots are unique robots embodied in the environment. Also, as they mimic neural processing and most are morphologically similar to humans, they can be used to test brain theories in the laboratory and real-life settings while serving alongside human team members (Krichmar and Hwu, 2022). Policies around neuro-robots would thus need to be more nuanced and detailed. For example, how can we deploy a neuro-robot safely in a dynamic environment to enhance our understanding of neuroscience? Relatedly, can we deploy neuro-robots in a natural setting without any ethical approval in the first instance? Furthermore, as neuro-robots are complex and rely on enhanced human-robot interaction, how do we ensure that neuro-robots present a meaningful and trustworthy explanation in a human-friendly manner interpretable to both engineers and the public alike? Who will be held responsible if the neuro-robots go wrong, the neuro-robots or the developers? How do we gauge their performance in functions such as walking, singing, cooking, playing cards, or typing on keyboards? Do we benchmark their performance against human counterparts? Lastly, how much autonomy do we give them, and do we control them?

Modeled after biological brains, neuro-robots use a combination of neurally-inspired computing and AI. Neuro-robots operate on noisy and often uncertain data (Khan et al., 2022), to make decisions to help reduce the workload of fellow humans. When these robots work, they are of huge utility, allowing for, among other things, prosthetics (McMullen et al., 2014; Johannes et al., 2020; Handelman et al., 2022; Rejcek, 2022) and wearable systems supporting locomotion and learning processes (Colachis et al., 2018; Billard and Kragic, 2019). These systems can also engage in self-teaching modes allowing them to work like humans as waiters, deliverers, receptionists, troubleshooters, and digital assistants (Financial Times, 2022; Meta Fundamental AI Research Diplomacy Team (FAIR) et al., 2022).

However, as with human intelligence, sometimes robots fail to deliver, as mentioned earlier. In the instance of Henn na Hotel, the robots mistakenly took human snoring at night as a legit guest’s request, thus prompting them to disturb the guests at night. In other words, the robots could not perform feature extraction and intelligent comprehension of human snoring. The failure of robots is not startling. Intelligence is the act of decision-making based on prevailing uncertainty (Khan et al., 2022). This fact differentiates robots deploying AI from non-intelligent decision systems based on the flow-chart design, as is the case in most electronics (Friedman and Zeckhauser, 2012). For human beings, such failures are vital for learning during childhood and adulthood. Neuro-robots using machine learning (ML) AI algorithms also require a “training phase” whereby the system is first trained on a human-labeled dataset (MIT Technology Review, 2018). The system then learns from its failures before being permitted to operate in the “wild” (MIT Technology Review, 2018). Therefore, it is understandable that, despite training, humans and neuro-robots might mis-categorize a new data episode that had never been seen or used.

In the case of human intelligence, only recently has neuroscience offered a mechanistic picture of the cellular basis of learning and memory (Liu et al., 2012). However, for neuro-robots, explaining why failures occur is not readily explainable. This is in spite of the EU regulations and other guidelines requiring that robotic actions’ traceability and explainability be available to EU citizens to protect them from potential adverse effects.

For this paper, explainable neuro-robots are neuro-robots whose function and decision-making processes must be explained so that the average person can comprehend the basis of a robot behavior. Here we summarize the existing trends in neuro-robots and their underlying circuitry. Then we recap some literature surrounding failures neuro-robots may encounter. Since neuro-robots deploy AI and ML algorithms in their design, a summary of explainable AI methods and associated problems is provided in the following section. Finally, we will offer themes of how the explainability of neuro-robots may be researched as the field advances further.

## 2. Advances in neuro-robotics and robotic failures go hand in hand

While much of the basis for human higher cognition remains unknown, the existing understanding of biological brains deployed in biomimetic fashion in embodied intelligence, developmental robotics, and AI inform ways to design intelligent robot systems. Here we term these systems neuro-robots. Neuro-robots imitate aspects of the human brain in their design and function (Krichmar, 2018). Such robots can also interact with the nervous system of humans or other animals (Iosa et al., 2016). Neuro-robots differ from other electromechanical devices based on the ability to adapt their behavior on the basis of their experience (Iosa et al., 2016), a characteristic that has been termed “adaptability.” Adaptability, in turn, is based on its multiple sensors, whose signals are processed by AI to change the robot’s behavior (Iosa et al., 2016).

A neuro-robot can be designed for clinical uses, for instance, neurorehabilitation or neurosurgery (Nordin et al., 2017). They can also be developed for studying the nervous system by mimicking its properties (Krichmar, 2018; Chen K. et al., 2020), as happens in many walking robots based on central pattern generators (Ijspeert et al., 2007).

Immersed in the real world, a neuro-robot takes sensory information from the environment before integrating it into actions (Chen K. et al., 2020). This information intake and resulting computation inform how the artificial brain gives rise to new behaviors based on its experience. Researchers have used neuro-robotic approaches to study the neural correlates of visual perception (Priamikov et al., 2016), tactile perception (Pearson et al., 2007), auditory perception (Rucci et al., 1999), spatial navigation (Lambrinos et al., 2000), schema formation and consolidation (Hwu et al., 2020), neuromodulation (Sporns and Alexander, 2002), attention (Gigliotta et al., 2017), locomotion (Lock et al., 2013), language development (Oudeyer, 2006), and social interaction (Boucenna et al., 2014). Readers interested in how the behavior of neuro-robots help explain their neural control and the analysis of how neural activity leads to behavior may further refer to seminal research (Chen K. et al., 2020).

Neuro-robotic systems have advanced substantially in functions and properties because of significant progress in brain-inspired computing algorithms and hardware (Krichmar, 2018). On the functionality front, most neuro-robots perform single tasks in simple and static situations for now (Zou et al., 2020). But, then, a few multitasking robots perform in dynamic environments (Zou et al., 2020). As for properties, current neuro-robots exhibit features ranging from intelligent perception and flexible movement to interactions with environments (Sanders and Oberst, 2016).

The critical element of the software stack for neuro-robots is brain-inspired computing algorithms, which witnessed tremendous advancement in the past decade. Of those algorithms, two main categories are Artificial Neural Networks (ANNs) and Spiking Neural Networks (SNNs). While the human brain’s hierarchical topologies and parallel-processing networks inspire ANNs (Yang and Wang, 2020), SNNs take inspiration from the patterns of neuronal action potentials subserving human brain function (Ghosh-Dastidar and Adeli, 2009). ANNs, specifically deep neural networks, are lauded for their phenomenal success in various machine-learning tasks (Zou et al., 2020). For instance, deep neural networks have already achieved human-level performance in image recognition (van Dyck et al., 2021). While most traditional robots use backpropagation to train ANNs (Hecht-Nielsen, 1989), neuro-robots usually model the ANNs around neuro-anatomically grounded Hebbian learning rules and algorithms (Garagnani et al., 2008). Similarly, SNNs are also very powerful computing and highly energy-efficient paradigms for processing dynamic sequential information (Cao et al., 2015; Zou et al., 2020). These paradigms possess desirable features such as high bio-fidelity, rich coding with complex data, and event-driven idiosyncrasy (Bichler et al., 2012; Zou et al., 2020).

Other important brain-inspired algorithms for robots include attractor neural networks (Solovyeva et al., 2016; Khona and Fiete, 2022). Such networks are recurrent dynamic networks, evolving toward a stable pattern (either single state, cyclic state, chaotic state, or random state) over time (Khona and Fiete, 2022). Attractor networks typically model neuronal processes such as memory, motor behavior, classification, and other biologically inspired processes in machine learning (Li et al., 2015).

The advancement of algorithms goes hand in hand with the development of neural computing hardware, also called neuromorphic architectures. On the one hand, we have neural network accelerators that optimize operations in ANNs and usually leverage parallel processing and efficient data compression (Zou et al., 2020). Examples of such accelerators include ShiDianNao (Du et al., 2015) and TPU (Jouppi et al., 2017). On the other hand, neuromorphic chips are designed to support rich spatiotemporal bio-functionality (Zou et al., 2020). Such chips provide high energy efficiency and event-driven representations. Examples include Neurogrid (Benjamin et al., 2014), SpiNNaker (Furber et al., 2013; Krichmar et al., 2019), IBM’s TrueNorth under the SyNAPSE project (Merolla et al., 2011; Modha et al., 2011; DeBole et al., 2019), and the energy-aware computing hardware developed by HRL laboratories (Srinivasa and Cruz-Albrecht, 2012). Alongside neural accelerators and neuromorphic chips, we have many human-inspired robotic hardware (in humanoid platforms) that provides a maximum degree of anatomical fidelity to the human structure and is capable of whole-body motions (ECCE Robots, 2010; Ackerman, 2016b; WIRED, 2017). Examples of these platforms include ECCE (ECCE Robots, 2010; Bonsignorio, 2013), Kengoro (Ackerman, 2016b; WIRED, 2017), and HRP-2 (Hirukawa et al., 2004), among others.

These breakthroughs in algorithms and hardware led to the development of advanced neuro-robots that exhibit intelligent perception and flexible movement (Krichmar, 2018). Regarding functionality, we have two developmental designs: single-task robots and multitask robots (Zou et al., 2020). Single-task robots operate in simple scenarios with limited capability to perform multiple functions. On the other hand, multitask robots navigate dynamic systems and can perform multiple tasks simultaneously. Both these types of robots and systems are prevalent in different real-world applications, such as medical robots (Davies et al., 2000; Chen A. et al., 2020), prosthetic arms (Fazeli et al., 2019), humanoid platforms (Johansson et al., 2020), and automated driving (Spielberg et al., 2019). These applications offer critical opportunities to advance the design of robot systems further.

Neuro-robots deploying non-von Neumann architectures, specifically neuromorphic engineering, can provide low-power processing (on the order of milliwatts or watts, compared to kilowatts for a GPU) and sensing for autonomous systems. For example, the TrueNorth neuromorphic chip of IBM has deployed convolutional neural networks (CNNs) on autonomous robots and other embedded applications with minimal power consumption (Esser et al., 2016; Hwu et al., 2017). Similarly, neuromorphic architectures enable next-generation processing (Indiveri et al., 2011; James et al., 2017). Furthermore, by incorporating a processor-in-memory and event-based design, neuromorphic processors can provide three orders-of-magnitude strategic advantages in performance-per-watt while being robust to radiation effects.

While substantial effort has been invested in making robots more reliable in terms of power processing and dynamic sensing, experience demonstrates that frequent failures often challenge robots. According to researchers, the mean time between failure (MTBF) for robots in field environments is usually within a few hours (Tsarouhas and Fourlas, 2016). Regardless, robots have yet to reach a design that can better cater to fault management (Honig and Oron-Gilad, 2018). Even trained roboticists are not entirely aware of what causes the failure (Steinbauer, 2013). There is a plethora of literature on robotic failures (Laprie, 1995; Carlson and Murphy, 2005; Steinbauer, 2013; Barakova et al., 2015; Lemaignan et al., 2015; Brooks, 2017; Honig and Oron-Gilad, 2018). Failures refer to a degraded ability that causes the system’s behavior or service to deviate from the standard or correct functionality (Honig and Oron-Gilad, 2018). Various errors and faults can cause failures in systems. For instance, the Henn na robots experienced failure due to speech-recognition errors. Similarly, in the autonomous car accident, the car crashed into a white truck due to intelligent feature extraction problems (Shepardson, 2017). Finally, Pepper and other humanoid robots failed due to weaker system-environment interaction and unreadiness to handle unplanned situations, among other related issues (Pfeifer and Bongard, 2007).

While failure detection and fault prediction techniques and algorithms in neuro-robots are still emerging, literature discusses failure detection and prediction in traditional robotic controls and manipulators. Several algorithms and methods can accomplish this: second-order sliding mode algorithm (Ferrara and Capisani, 2012), robust non-linear analytic redundancy technique (Halder and Sarkar, 2007), partial least square approach (Muradore and Fiorini, 2012), torque filtering and sensing technique (Fu and Cai, 2021), multiple model adaptive estimation method (Akca and Efe, 2019), multiple hybrid particle swarm optimization algorithm to realize multiple predictions failures (Ayari and Bouamama, 2017), and neural network for prediction of robot execution failures (Diryag et al., 2014). Identifying and understanding failures through the means mentioned are crucial in designing reliable robots that return meaningful explanations to users when and if needed. At the same time, we agree it may be impossible to identify (or predict) all sorts of robotic failures as robots operate in dynamic and unorganized environments interacting in numerous possible ways. This becomes even more challenging for neuro-robots situated in natural settings with many unplanned events. However, several researchers have advanced insightful failure classifications that may also be well relevant for neuro-robots.

Some researchers classified robotic failures into technical failures and social norm violations (Giuliani et al., 2015). Technical issues inside the robots cause technical failures. In contrast, social norm violations refer to inappropriate social cues, for instance, robots looking away from a person while talking to them. Other researchers categorized failures according to the source of the failure (Carlson and Murphy, 2005). Such classification considers physical failures (physical errors in the system’s sensors, control system, or communications cause such failures) and human failures (human-made errors in design, for instance, cause these failures). Using information such as relevance (if the fault is relevant to various robotic systems), condition (context of failure), symptoms (indicators to identify the failure), impact (repairable or terminal, for instance), and frequency (how often it occurs), Steinbauer classified failures in two four categories following the RoboCup competitions: Interaction, Algorithms, Software, and Hardware (Steinbauer, 2013). Interaction failures arise from uncertainties in interacting with the environment and humans, whereas algorithmic failures are problems in methods and algorithms. Similarly, software failures are due to the design and implementation faults of software systems, whereas hardware failures are physical faults of robotic equipment.

Furthermore, Brooks classifies failures into communications and processing failures (Brooks, 2017). Communication failures are related to data being processed, including missing data (incomplete data), incorrect data (data distorted during transmission, for example), bad timing (data received too early or late), or extra data (for instance, data sent many times). Processing failures can happen due to poor logic (based on incorrect assumptions), ordering (when events occur in a different order), or abnormal termination due to unhandled exceptions or segmentation faults.

Other researchers echo some of these classifications and devise more inclusive human-robot failure categorizations (Honig and Oron-Gilad, 2018). Per their classification, there are two types of failures: technical (that includes both software and hardware) and interactive failures that arise because of uncertainties while interacting with the environment or agents in the environment, including humans. While all types of failures are important, interactive and algorithmic failures seem even more pertinent to neuro-robots serving and working alongside humans.

To use Laprie’s words (Laprie, 1995), some of these failures are “catastrophic”—with a higher cost than the service—and thus, they need to be avoided. To avoid failures, the failures need to be understood and explained. The explanation for most of these failures, particularly the ones relating to interaction and algorithms, is not readily available. An explainable neuro-robot will be expected to unravel some of this explanation to end users and engineers alike.

## 3. Explainability in AI limits explainability in neuro-robots

Neuro-robots are useful but still not explainable as they employ a combination of AI algorithms and neural-inspired hardware while interacting with environments and agents in evolving settings. In addition, as an interdisciplinary field, neuro-robotics involves control and mechatronics, among other areas. Although in the previous section, we noted some failures and failure detection techniques regarding the control, design, and engineering, we limit ourselves to algorithms for the purpose of this discussion. The challenges inherent in neural-inspired hardware are attributed to the fact that neuroscience itself lacks a complete theory of brain function, which is further compounded by the sheer physical complexity of biological brains (10^15^ computational synaptic elements in the human brain). For AI, the explanation of why failures happen is not easily available (Gunning and Aha, 2019). Explainability in neuro-robots is thus largely limited by the explainability in AI. However, robotic (neuro-robotic) failures identified earlier warrant explanation as they impact lives and livelihoods in many consequential ways. We propose as AI explainability increases, neuro-robots will become more explainable. Thus, this section draws an overview of current explainable AI methods, which provide a foundation for explainable neuro-robots.

Machine learning (ML) explainable techniques (or X-AI methods) offer an understanding and explanation of ML models’ decisions in human terms to establish trust with stakeholders, including engineers, users, and policymakers. In the past decade, with the application of AI in several autonomous systems and robots, we have seen a tremendous amount of research interest in X-AI methods. Currently, we can choose from a suite of X-AI methods to untangle deep learning opaque models (Lipton, 2017; Došilović et al., 2018; Xu et al., 2019; Holzinger et al., 2022; Khan et al., 2022). There are various categorizations of X-AI methods based on several criteria, including structure, design transparency, agnostic-ness, scope, supervision, explanation type, and data type, as listed in Table 1 (Khan et al., 2022).

**TABLE 1:** Summary of existing explainable AI (X-AI) methods.

Criterion	Types	Definitions	Examples
**Structure** Relates to the complexity of ML models	Intrinsic (Xu et al., 2019; Holzinger et al., 2022)	Easily interpretable model because of their simple structure	Linear regression, logistic regression, decision trees, and k-nearest neighbors
	*Post hoc* (Madsen et al., 2022; Yera et al., 2022)	Complex structure models that attain interpretability after model training.	Permutation feature importance and neural networks
**Transparency in design** Relates to the design of a method	Whitebox (Levashenko et al., 2016; Garibaldi, 2019; Loyola-González, 2019; Zaitseva et al., 2020)	By design, Whitebox approach is more transparent and explainable.	Simple decision trees, rule-based models, patterns-based models, linear regression models, bayesian networks, fuzzy cognitive maps, and those following fuzzy logic such as fuzzy decision trees and fuzzy rules-based models
	Blackbox (Robnik-Šikonja and Kononenko, 2008; Loyola-González, 2019)	Blackbox approach contains complex mathematical functions like support-vector machine and neuronal networks. Generally, they are hard to understand and explain.	Deep neural networks and random forests
	Greybox (Pintelas et al., 2020)	Such approaches have features of both Blackbox and Whitebox approaches.	Local Interpretable Model-agnostic Explanations (LIME) and Interpretable Mimic Learning
**Scope** Relates to the scope of the interpretability	Local (Ribeiro et al., 2016; Lundberg and Lee, 2017)	The scope of interpretability is limited to individual predictions or a small portion of the model prediction space.	Local Interpretable Model-agnostic Explanations (LIME), SHapley Additive exPlanations (SHAP), and Individual Conditional Expectation (ICE)
	Global (Lundberg et al., 2020; Machlev et al., 2022)	Global methods cover the entire model prediction space.	Partial Dependence Plot (PDP) and Accumulated Local Effects
**Agnosticity** Classification based on the level of agnostic-ness	Model agnostic (Machlev et al., 2022)	Their X-AI algorithm can be applied to any kind of ML model. They do not depend on model internals.	SHAP and LIME
	Model specific (Belle and Papantonis, 2021)	Methods are designed for specific types of ML model.	Neural network methods
**Supervision** Classification based on the degree of supervision	Supervised (Ancona et al., 2019)	Entail an active manipulation of input data.	LIME, SHAP, integrated gradients, smoothgrad, layer-wise relevance propagation, and perturbation methods
	Unsupervised (Lei et al., 2016; Esmaeili et al., 2019)	Researchers assume no explicit annotations about input data.	Rationale and disentanglement representations
**Explanation or data type** Additional methods based on type of explanation or data	Explanation type (Belle and Papantonis, 2021; Holzinger et al., 2022)	Explanation output differs in each method. For instance, feature summary return feature statistics.	Feature summary, surrogate models, extract concepts, decision rules, correlation plots, and other visualizations
	Data type (Welling et al., 2016; Hendricks et al., 2018; Pawelczyk et al., 2020)	Classification based on the data type a method can handle.	Graph, image, text/speech, and tabular

Most popular methods include intrinsic vs. *post hoc*, Blackbox vs. Whitebox vs. Graybox approaches, local vs. global approaches, model agnostic vs. model specific approaches, supervised vs. unsupervised methods, and those methods that differ in explanation type or the data type they can handle.

Overall, these excellent foundational methods [summarized in Table 1; for more details, readers may consult (Khan et al., 2022)] help produce some model understanding and present bits of human interpretable understanding. However, there is still no comprehensive understanding of how an AI implements a decision while explaining the model decision (Khan et al., 2022). These methods are far from perfect. High-stake ML deployment failures from these Blackbox models underpin the idea that these models fail to offer a satisfactory level of explanation (Ackerman, 2016a; Strickland, 2019; Su et al., 2019). The failures further underscore that these models are uncontestable, opaque, display unpredictable behavior, and in some situations, boost undesirable racial, gender, and demographic biases (Newman, 2021). Consequential settings such as healthcare, criminal justice, and banking have already witnessed substantial harm because of the issues found in Blackbox methods (Newman, 2021). Whitebox models, on the other hand, are also not the best: while these models are more interpretable, they are less accurate (Loyola-González, 2019).

Beyond an incomplete explanation, the explanation is unstable (Bansal et al., 2020; Kozyrkov, 2021). For instance, four X-AI methods were deployed to determine what makes a matchstick a matchstick (Bansal et al., 2020). By changing only a single parameter, the methods returned twelve explanations, suggesting the methods are unstable (Choi, 2021). Even state-of-the-art techniques such as Local Interpretable Model-agnostic Explanation (LIME) and Shapley Additive exPlanations (SHAP) deploying Local Linear Explanations (LLE) also suffer from defects, including unstable explanations after changing a single parameter or even different explanations for the same data point (Amparore et al., 2021). As these methods use some randomized algorithm, for example, the Monte Carlo algorithm, to explain decisions and predictions—like deep neural networks mislabeling the image of a lion as a library (Szegedy et al., 2014) or AI failing to predict a husky on the snow (Ribeiro et al., 2016), or even AI taking a horse for a frog (Su et al., 2019)—the resulting explanation may vary (Amparore et al., 2021).

The variability and heterogeneity in explanations stem largely from model dynamics, algorithms, and internal mechanics. Beyond these issues, X-AI methods suffer from external validity issues and cannot handle all sorts of data and environments. The validity issue is even more severe when the methods employed to the same data generate different predictions, as mentioned in the example of matchstick prediction (Bansal et al., 2020). Such external validity issues will be a matter of deep concern with the increasing application of AI to human ML systems in healthcare, education, justice, defense, and security.

A recent article comprehensively outlines the multidimensional challenges faced by X-AI methods (de Bruijn et al., 2022). Some of these issues pertain to data and decision dynamics (varying data and decisions lead to different explanations) and dependency on context (since outcomes may differ for various individuals, general explanations for algorithms may not work). Other challenges speak to the “wicked” nature of the problems (the poorly designed nature of the problems requires multiple answers versus a single answer that current algorithms furnish) and the contested nature of explanations due to biasedness, among other concerns.

As neuro-robots get deployed in social settings and encounter obstacles and humans, they will most likely make mistakes in their activities, such as walking (neuro-robotic navigation). To reiterate, the explainability of neuro-robots’ behavior is needed, and even more so from a safety perspective, as we do not want humans to get scared or hurt by neuro-robots. When applied to neuro-robots, some X-AI methods, such as LIME, SHAP, data type-based methods, and neural networks, can offer initial insights into neuro-robotic behavior just like they provided explanations in traditional robotics. Earlier work on robots produced verbal and natural language explanations (data-based explanations) of robotic navigation (Perera et al., 2016; Rosenthal et al., 2016; Stein, 2021). Such works deployed algorithms that translated sensor data into natural language. While these algorithms are interpretable, the entire narration of trajectory may not be appealing. Unlike natural language explanation, Halilovic and Lindner (2022) offer visual explanations using LIME (taking image data as input) to explain deviations from desired navigation path. While we know how LIME explains predictions of a Blackbox model by learning an interpretable model around a deviation (Halilovic and Lindner, 2022), LIME takes more time to generate an explanation (Halilovic and Lindner, 2022) as well as performs better when generating an explanation for a single prediction (Banerjee, 2020). Thus, LIME perhaps cannot be used solely to generate multiple and varying explanations needed from neuro-robots as they operate in fast-evolving complex environments. LIME, however, may be used down the road effectively once its runtime is improved. Also, this improved version of LIME may be used in conjunction with other methods, such as the Partial Dependence Plot (PDP), to generate an explanation of the entire model in neuro-robotics.

Other researchers use neural networks (specifically, deep reinforcement learning) (He et al., 2021) or fuzzy algorithms (Bautista-Montesano et al., 2020) to explain robotic navigation. But, again, while these are great methods, the former explanation is too complex for non-specialists, whereas the latter explanation makes a strong assumption about the underlying algorithm.

Relatedly, many researchers have used model-agnostic X-AI methods to enhance expert understanding of deep learning Blackbox models and their outputs generated in traditional robots (Raman and Kress-Gazit, 2013; Adadi and Berrada, 2018; Rai, 2020); other studies used methods that focused only on classification-based tasks (Ribeiro et al., 2016; Wu et al., 2017), including the application of saliency map by Huber et al. to improve the interpretation of image classification tasks (Huber et al., 2022) and feature extraction to determine the effect of an individual feature on utility function (Elizalde et al., 2008). As opposed to these studies and to account for sequential decision-making that robots perform, researchers employed a framework for generating linguistic explanations from robot’s state sequences using an encoder-decoder model (Ehsan et al., 2019; Das et al., 2021). Whereas the former research studies generate primarily expert-centric explanations, the latter user-centric studies seem more promising in the context of neuro-robots as they account for the complexity of sequential decision-making carried out by such robots while interacting with users and objects in the environment.

In real life, neuro-robots will encounter multiple challenges and obstacles. Aside from deviations in navigation and task classification in static settings, there will be other collapses and breakdowns in dynamic environments. Also, the neuro-robots will be challenged to generate meaningful explanations using real-time data and recall a memory from past experiences. In addition, they will face ethical dilemmas. While the present trajectory of X-AI methods holds great promise for the design of transparent robots in labs, they are not yet powerful enough to fully incorporate explanations for unexpected events, instances, and failures.

The issues, from unstable to complex explanations and those around data and decision dynamics mentioned in this section, limit the explanatory power of neuro-robots, particularly those deployed in social settings facing unexpected hiccups and situations. As these embodied neuro-robots employ AI to sense and act on their environment, and AI’s outcome explanation is deficient, neuro-robots will lack the explanation required by users, engineers, and policymakers alike. Thus, to make neuro-robots more explainable, one way researchers can accomplish this goal is to develop a robust neural framework that explains the AI outcome to satisfaction, as suggested by the authors in their recent paper (Khan et al., 2022).

In its design, such a neural framework will be inspired by biological mnemonic function to produce an explanation. In biological brains, the members of a cell assembly, by their act of firing action potentials together, are involved in memory formation (i.e., an engram)—this essentially constitutes an explanation. The active cells hence identified are then deactivated optogenetically to reversibly control the recall of the specific memory (Liu et al., 2012). This mnemonic function is considered a particular instance of decision-making since each decision requires a corresponding memory. The neural framework mentioned here, while still needing to be tested, nevertheless provides a way forward for offering a fuller explanation. If successful, neuro-robots may also deploy this framework to offer explanations in the events they are required to give one.

## 4. Toward explainable neuro-robots

Traditional AI systems are evolving exponentially. Some of these systems engage in self-teaching modes that permit them to surpass human capabilities at games like chess and Go (Silver et al., 2016, 2017) and, most recently, Diplomacy (Buch et al., 2022; Financial Times, 2022; Meta Fundamental AI Research Diplomacy Team (FAIR) et al., 2022). Moreover, neuromorphic architectures are also undergoing further advances (DeBole et al., 2019; Olds, 2019). New chips, such as Intel’s Loihi, are being developed to support embedded neuromorphic applications (Davies et al., 2018). In addition to running neural networks on specialized hardware, very low-power neuromorphic vision and auditory sensors are being developed (Liu and Delbruck, 2010; Stewart et al., 2016). Similar to biology, these sensors and processors only respond to change or salient events. When they do respond, it is with a train of precisely timed spikes, like a neurobiological system. The event-driven nature leads to ideal power efficiency for autonomous systems and robots.

With all these new advances and developments, neuro-robots will further mature in design and function. However, increasing instances of robotic failures require neuro-robots to furnish an explanation that uses real-time data and previous experiences. Moreover, since neuro-robots rely massively on human-robot interactions, the explanation would have to be trustworthy and offered in a friendly manner. Finally, as outlined earlier, legislative pressures in Europe and America alongside IEEE guidelines make the design and use of explainable neuro-robots and the emerging explanation even more urgent. Thus, researchers designing neuro-robots must ensure that robotic design and deployment integrate explanatory capability as a core principle.

Accomplishing the next steps will not be easy. The ecosystem of jurisdictions that might adopt appropriate policies to accomplish the above is incredibly diverse, ranging from local to transnational entities and even including Outer Space. Further, there is no current consensus within the neuro-robotics community. Finally, as we have outlined above, the ontology of the field itself is complex. Nevertheless, there are models that might be useful to consider. One of those involves the regulation of pharmaceuticals and medical devices across international boundaries. As with neuro-robotics, the potential to do harm is significant. Additionally, there are important external Incentives for the coordination of policies (e.g., COVID-19 vaccines). Therefore, we recommend creating an international body under the auspices of the United Nations to coordinate a “policy bridge” between researchers and international stakeholders analogous to the World Health Organization (WHO). Such an organization could both catalyze the development of uniform standards and language ontologies relevant to the problem and draft model regulations that jurisdictions might adopt either in whole or in part.

In conclusion, we have reviewed the current state of play in the field of X-AI within the context of neuro-robotics. Because of the significant consequence of robotic failure on human (and even planetary) welfare, it is imperative to move forward, notwithstanding the challenges and complexity of the field.

## Author contributions

Both authors listed have made a substantial, direct, and intellectual contribution to the work, and approved it for publication.
